# Development of a Nomogram for Moderate-to-Severe Bronchopulmonary Dysplasia or Death: Role of N-Terminal Pro-brain Natriuretic Peptide as a Biomarker

**DOI:** 10.3389/fped.2021.727362

**Published:** 2021-08-23

**Authors:** Min Song, Mengyuan Lei, Chenghan Luo, Zanyang Shi, Xinru Cheng, Wenqian Ding, Wenjun Cao, Jingdi Zhang, Jian Ge, Mengmeng Wang, Peige Xia, Fengxia Mao, Li Wang, Qian Zhang

**Affiliations:** ^1^Neonatal Intensive Care Unit, The First Affiliated Hospital of Zhengzhou University, Zhengzhou, China; ^2^Health Care Department, The First Affiliated Hospital of Zhengzhou University, Zhengzhou, China; ^3^Orthopeadics Department, The First Affiliated Hospital of Zhengzhou University, Zhengzhou, China

**Keywords:** bronchopulmonary dysplasia, N-terminal pro-brain natriuretic peptide, biomarkers, nomogram, preterm infants

## Abstract

**Objectives:** This study aimed to explore the clinical value of N-terminal pro-brain natriuretic peptide (NT-proBNP) in predicting moderate-to-severe bronchopulmonary dysplasia (BPD)/death, and to establish an effective clinical predictive nomogram.

**Methods:** We retrospectively analyzed very low birth weight infants (VLBWs) with gestational age ≤ 32 weeks. The NT-proBNP values were determined on the 1st, 3rd, 7th, 14th, 21st, and 28th days after birth. The correlation between NT-proBNP level and moderate-to-severe BPD/death was evaluated. Receiver operating characteristic (ROC) curve analysis was used to evaluate the prediction ability. Then, we used multivariable logistic regression to build the prediction model and nomogram, and calibration of the model was assessed by calibration curve.

**Results:** In total, 556 VLBWs were involved, among whom 229 developed BPD (mild: *n* = 109; moderate: *n* = 68; severe: *n* = 52) and 18 died. The NT-proBNP level in the moderate-to-severe BPD/death group was significantly higher than that in the no-to-mild BPD group from the 3rd to 28th day (*P* < 0.001). When the natural logarithm of the serum NT-ProBNP level increased by 1 unit at day 7 (±2 days) of life, the risk of moderate and severe BPD/death was the highest (OR = 3.753; 95% CI: 2.984~4.720), and ROC analysis identified an optimal cutoff point of 3360 ng/L (sensitivity: 80.0%; specificity: 86.2%; AUC: 0.861). After adjusting for confounding factors, the level of NT-proBNP at day 7 (±2 days) of life still had important predictive value for the development of moderate-to-severe BPD/death, significantly improving the predictive ability of the model.

**Conclusion:** The level of NT-proBNP at day 7 (±2 days) of life can be used as an early promising biomarker for VLBWs to develop moderate-to-severe BPD/death. We constructed an early predictive nomogram to help clinicians identify high-risk populations.

## Introduction

Bronchopulmonary dysplasia (BPD) is a serious chronic pulmonary morbidity in childhood and is one of the most common and serious sequelae of prematurity (1). The incidence of BPD in very low birth weight infants (VLBWs) is between 11 and 50%, which is negatively correlated with gestational age (GA) and birth weight (BW) and varies considerably with different diagnostic criteria (2–4). With the improvement of neonatal care techniques, more premature infants, particularly VLBWs, can survive, leading to an increase in the incidence of BPD (5). Compared with patients with no-to-mild BPD, moderate-to-severe BPD affects survival and neurodevelopment, hospitalization is longer, mortality is higher, and it may be accompanied by a heavy life burden (6, 7). Presently, the evaluation time of BPD classification is 36 weeks postnatal menstrual age (PMA) or discharge to home, a time that is late and difficult for early intervention (8). Therefore, identifying predictors that can identify high-risk infants with moderate-to-severe BPD early is a critical research field.

N-terminal pro-brain natriuretic peptide (NT-proBNP) is a low-molecular-weight peptide (8.5 kDa) secreted by cardiomyocytes in response to volume overload (9). Compared with brain-type natriuretic peptide (BNP), NT-proBNP has a more stable chemical structure *in vitro* and can remain stable in blood samples for at least 72 h (10). Sellmer et al. (11) reported that NT-proBNP levels on day 3 were associated with BPD or death in a study of 183 premature infants whose GA was <32 weeks. In another small study (*n* = 51) of GA <30 weeks, Harris et al. (12) found that the level of NT-proBNP at 10 days after birth was valuable in predicting severe BPD. Additionally, Khan et al. (13) showed that the NT-proBNP levels on the 28th day after birth were moderately predictive of the severity of BPD in a prospective study. These studies all measured the NT-proBNP levels on a particular day, but the postnatal NT-proBNP levels in newborns are a dynamic process. Our study had a larger sample size and dynamically measured NT-proBNP levels within 28 days of birth, features that can better evaluate the value of the serum NT-proBNP levels as an early biomarker of BPD severity or death. Thus, a convenient and effective clinical predictive model including clinical features and biochemical indicators can be established that may help high-risk children obtain effective individualized treatment in the early stage to avoid the occurrence of moderate-to-severe BPD/death as much as possible.

## Materials and Methods

### Patients and Study Design

This retrospective study included premature infants delivered at the First Affiliated Hospital of Zhengzhou University and admitted to the Neonatal Intensive Care Unit (NICU) from November 01, 2016 to February 28, 2021 with GA ≤ 32 weeks and BW ≤ 1,500 g. Infants with congenital metabolic defects or malformations, complex congenital heart disease [except patent foramen ovale (PFO) or patent ductus arteriosus (PDA)], severe renal insufficiency, death in the first week of life and incomplete general data were excluded. This study was approved by the Medical Ethics Committee of the First Affiliated Hospital of Zhengzhou University (Ethical code: 2019-KY-95).

### Data Collection and Definitions

The data were collected from the maternal and infant medical records and included prenatal factors such as maternal age, hypertensive disorder complicating pregnancy (HDCP), gestational diabetes mellitus (GDM), hypothyroidism with pregnancy, the use of prenatal steroids, premature rupture of membranes (PROM), fetal distress and intrauterine growth restriction (IUGR), perinatal factors such as GA, BW, sex, assisted reproductive technology, mode of delivery, 1- and 5-min Apgar scores, neonatal respiratory distress syndrome (RDS), hemodynamically significant patent ductus arteriosus (HsPDA) and pulmonary hypertension (PH). At the same time, the use of alveolar surfactant and caffeine and the time of mechanical ventilation (MV) in the first week of life were recorded. According to the definition of NICHD, the diagnostic criterion of BPD is that patients receive oxygen therapy with an oxygen concentration >21% for at least 28 days, and are divided into no, mild, moderate or severe BPD groups according to the mode of respiratory support and concentration of supplementary oxygen at 36 weeks PMA (14).

Some perinatal risk factors in this paper were defined as follows: HDCP included preeclampsia, eclampsia, and gestational and chronic hypertension, which was defined as systolic blood pressure ≥ 140 mmHg or ≥ 90 mmHg during pregnancy (15). Hypothyroidism with pregnancy was defined as the level of serum thyrotropin exceeding the upper limit of the reference range and level of serum-free thyroxine 4 lower than the lower limit of the reference range during pregnancy (16). Fetal distress was clinically defined as the presence of long-term deceleration, repetitive moderate-to-severe variable deceleration or repetitive late deceleration (17). IUGR was defined as an estimated fetal weight or abdominal circumference less than the 10th percentile of GA (18). RDS was defined as a clinical symptom of early neonatal respiratory distress with consistent chest radiologic features and requiring oxygen supplementation within 24 h after birth to maintain a saturation of more than 85% (19). HsPDA was defined as the ductal diameter exceeds 1.5 mm plus echocardiography at 7 days (±2 days) after birth shows predominant left to right flow, and need for ventilator support with symptoms and signs suggestive of symptomatic PDA including respiratory deterioration, cardiac murmur, precordial hyperactivity, oliguria, hypotension and widening pulse pressure (20). Early PH was assessed by measuring tricuspid regurgitation (TR) in echocardiography at 7 days (±2 days) and calculating right ventricular systolic blood pressure (RVSP) using the modified Bernoulli equation described by Skinner et al. (21).

### NT-proBNP Measurement and Collection

In the management of the NICU, our center attaches considerable importance to the monitoring of plasma NT-proBNP levels and cardiac function. For high-risk infants, the NT-proBNP levels were routinely measured on the 1st, 3rd, 7th, 14th and 28th days after birth. A small amount of venous blood samples was collected and centrifuged in a heparin lithium tube for 5 min (centrifugal radius = 6 cm, 3,500 r/min). Next, the samples were immediately detected using a fully automatic electrochemical immunoluminescence analyzer (Cobas 8000 Roche Elecsys 602; Roche, Switzerland) according to the instruction manual. The detection range of the concentration was 5–35,000 ng/L, and the coefficient of variation was 0.5%.

The serum NT-proBNP levels measured on the 1st, 3rd, 7th, 14th, 21st and 28th days after birth were collected, and the complete data on the 1st and 3rd days were collected. If the data collected on the 7th and subsequent days were not accurately measured at the sampling time, the measured values 2 days before or after the sampling time were approximately replaced. If multiple samples were taken at the same sampling time, the average NT-proBNP value was selected. The NT-proBNP value higher than the quantitative upper limit (>35,000 ng/L) was changed to the fictive value of 35,010 ng/L in this study. On the day of blood collection, we also collected data on the types of oxygen supplementation and respiratory support.

### Sample Size Estimation

In this study, 26 variables were analyzed by univariate analysis, and 14 variables were included in multivariate logistic regression. For binary endpoints, the rule of 10–20 events per variable (EPV) is generally followed to prevent overfitting (22). According to the principle of 20 EPVs per variable, this study required at least 280 EPVs. Our study included 556 preterm infants who met the criteria, so the sample size was sufficient.

### Statistical Analysis

Statistical analysis was performed using R software (Version 3.6.3; https://www.R-project.org). The quantitative data were tested for normality using the Shapiro-Wilk test. All the quantitative data in the study showed normal distribution. These data were expressed as medians (upper quartile, lower quartile) and were compared with the Mann–Whitney *U* test. The qualitative data were expressed as a percentage (%) and compared by χ2 test. Natural logarithmic transformation was used to improve the model fitting of NT-proBNP measurements. Univariate logistic regression analysis was used to evaluate the contribution of the NT-proBNP levels to the development of moderate-to-severe BPD/death. Receiver operating characteristic (ROC) curve analysis was used to evaluate the prediction ability, and the Jordan index was used to calculate the best cutoff point. Multivariate logistic regression was used to determine the final model, and the area under the curve (AUC) was used to evaluate whether the prediction ability of the model was improved in the presence of biomarkers. The nomogram of the model was established and verified internally using 1,000 bootstrap analyses. A calibration curve was plotted to evaluate the performance of the nomogram. Differences were considered statistically significant at *P* < 0.05.

## Results

During the study period, 581 premature infants with GA ≤ 32 weeks and BW ≤ 1500 g were admitted to the NICU of the First Affiliated Hospital of Zhengzhou University. Among those patients, 12 were excluded because of congenital metabolic defects, complex heart disease or severe renal insufficiency, five were not included because of death in the first week of life, and eight were excluded because of incomplete general data. Finally, 556 premature infants were included in the study. Among the participants, 82 participants were premature infants with GA ≤ 28 weeks. Additionally, 229 infants met the diagnostic criteria of BPD, including 109 mild, 68 moderate and 52 severe infants, and 18 died between 7 days after birth and 36 weeks PMA. We divided all the participants into two groups. The first group included infants with no-to-mild BPD (*n* = 418), and the second group included infants with moderate-to-severe BPD/death (*n* = 138). Compared with group 1, the patients in group 2 had more unfavorable baseline characteristics, such as lower GA, BW and Apgar scores, and were more likely to develop IUGR and fetal distress. Premature infants in group 2 were more likely to develop HsPDA and early PH (*P* < 0.05) (Table 1).

**Table 1:** Baseline characteristics and comparison of both study groups.

	**All patients**	**No-to-mild BPD**	**Moderate-to-severe BPD/death**	***P*** **-value**
	**(*n* = 556)**	**(*n* = 418)**	**(*n* = 138)**	
GA (weeks) [median (IQR)]	29.6(28.6,30.5)	30.0(29.1,31.0)	29.1(28.0,30.0)	<0.001
BW (g) [median (IQR)]	1,200 (1,050, 1,350)	1,250(1,100, 1,400)	1,100(920, 1,300)	<0.001
Male (%)	295(53.0)	207(49.5)	88(63.8)	0.004
Multiple pregnancy (%)	134(24.1)	100(23.9)	34(24.6)	0.865
Apgar score, 1 min [median (IQR)]	8(7,9)	8(7,9)	7(5.75,8)	<0.001
Apgar score, 5 min [median (IQR)]	9(8,10)	9(8,10)	8(7,9)	<0.001
Maternal age (years) [median (IQR)]	30(27,35)	30(27,35)	31(27,36)	0.471
HDCP (%)	254(45.6)	193(46.2)	61(44.2)	0.687
GDM (%)	83(14.9)	64(15.3)	19(13.8)	0.659
Hypothyroidism with pregnancy (%)	30(5.3)	21(5)	9(6.5)	0.5
History of abnormal pregnancy (%)	154(27.6)	106(25.4)	48(34.8)	0.032
Prenatal steroids (%)	170(30.5)	133(31.8)	37(26.8)	0.268
Assisted reproduction (%)	113(20.3)	81(19.4)	32(23.2)	0.335
Cesarean delivery (%)	431(77.5)	331(79.2)	100(72.5)	0.101
General anesthesia (%)	144(25.8)	107(25.6)	37(26.8)	0.778
PROM (%)	136(24.4)	108(25.8)	28(20.3)	0.189
Fetal distress (%)	120(21.5)	78(18.7)	42(30.4)	0.004
IUGR (%)	51(9.1)	28(6.7)	23(16.7)	<0.001
Use of surfactant (%)	452(81.2)	324(77.5)	128(92.8)	<0.001
RDS (%)	530(95.3)	397(95)	133(96.4)	0.499
HsPDA (%)	177(31.8)	112(26.8)	65(47.1)	<0.001
Early PH (%)	111 (20)	75 (17.9)	36 (26.1)	0.038

*BPD, bronchopulmonary dysplasia; GA, gestational age; BW, birth weight; IQR, Interquartile range; HDCP, hypertensive disorder complicating pregnancy; GDM, gestation diabetes mellitus; PROM, premature rupture of membranes; IUGR, intrauterine growth restriction; RDS, respiratory distress syndrome; HsPDA, hemodynamically significant patent ductus arteriosus; PH, pulmonary hypertension*.

**Figure 1:**
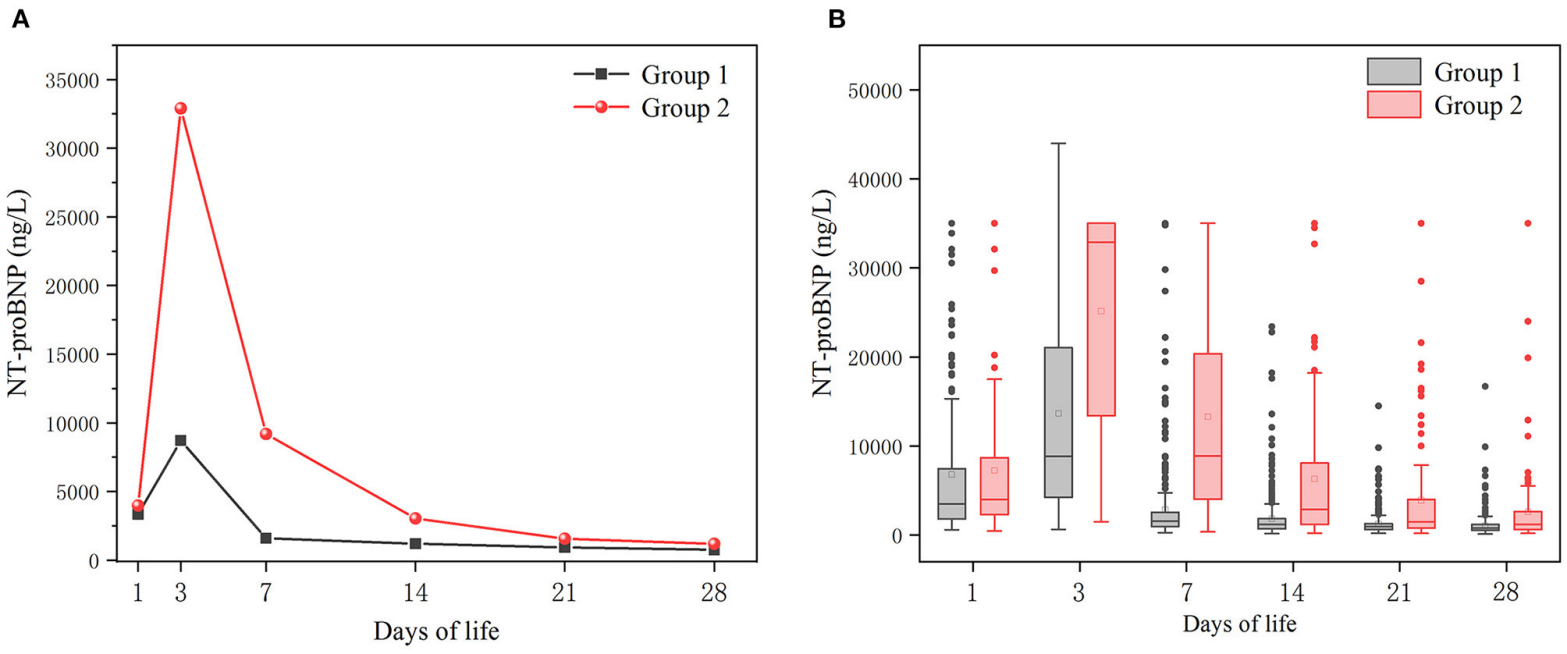
Temporal evolution of NT-proBNP levels in the two groups. Group 1: no-to-mild BPD group; Group 2: moderate-to-severe BPD/death group. **(A)** Median values of NTproBNP levels. **(B)** Box plot of NTproBNP levels.

**Figure 2:**
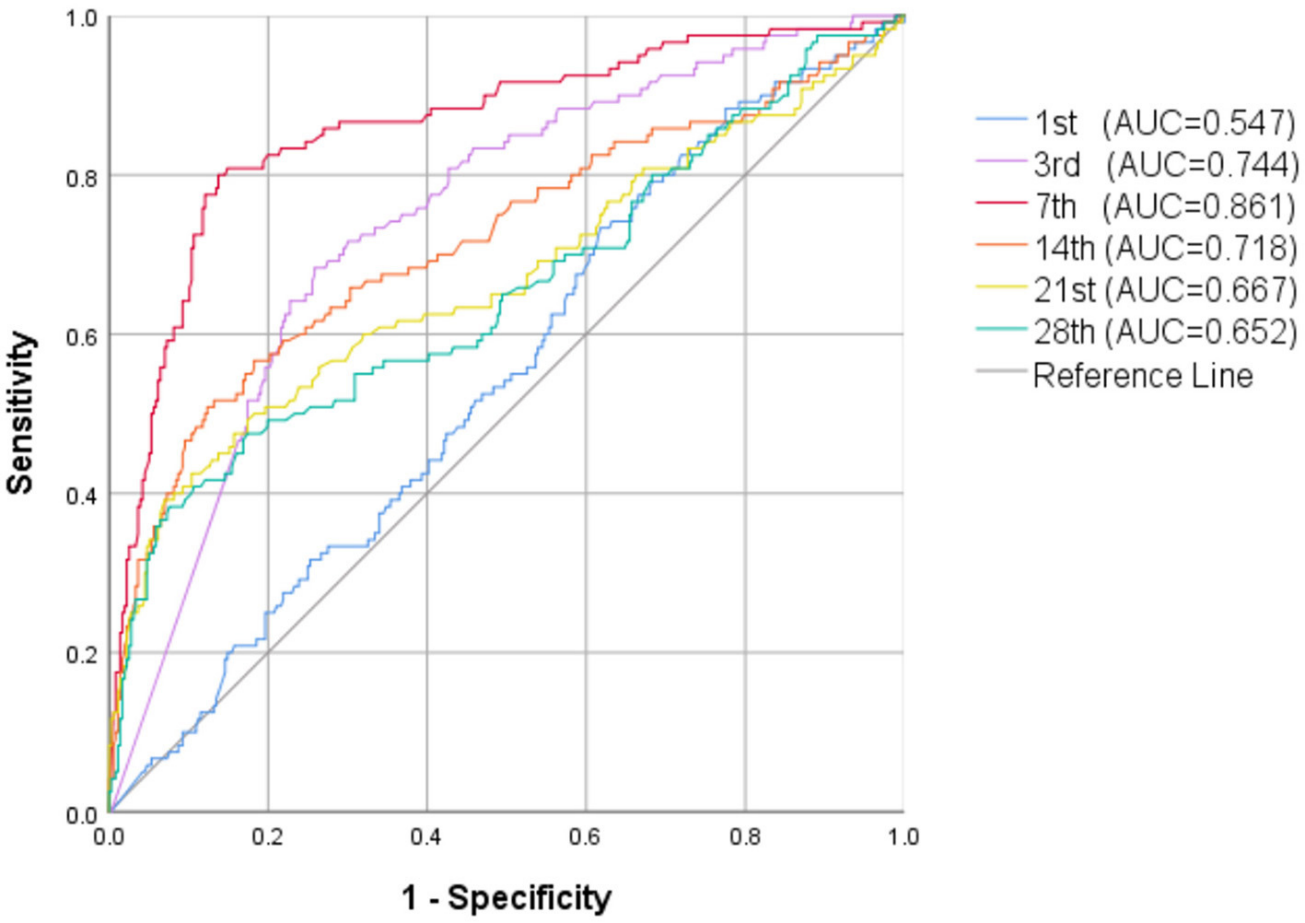
ROC curve of NT-proBNP levels in the first 28 days of life for prediction of moderate-to-severe BPD/death.

Whether in group 1 or group 2, the level of postnatal NT-proBNP showed an upward trend. The NT-proBNP value was at a high level on the 3rd day, and gradually decreased thereafter. On the 7th day (±2 days), the median (IQR) NT-proBNP levels of the two groups were 1590 (962–2,708) ng/L and 9185 (4010-23,975) ng/L respectively, and gradually tended to a stable low level on the 28th day (±2 days) (Figure 1). The NT-proBNP levels in group 2 were higher than those in group 1 from day 3 to day 28(±2 days) after birth (*p* < 0.001). When the natural logarithm of the serum NT-ProBNP level increased by 1 unit on the 7th day (±2 days), the risk of moderate-to-severe BPD/death was the highest (OR = 3.753; 95%CI: 2.984~4.720; *P* < 0.001; Table 2), and the predictive ability was the strongest. ROC curve analysis estimated that the AUC value was 0.861 (0.819~0.903), the best cutoff value of NT-proBNP on day 7 after birth (±2 days) was 3360 ng/L, the sensitivity was 80.0%, and the specificity was 86.2% (Figure 2).

**Table 2:** Univariate logistic regression of the association between NT-proBNP values (and IQR) during the first 28 days of life and moderate-to-severe BPD/death.

**Days**	**Num**	**All patients**	**No to mild BPD**	**Moderate to severe BPD/Death**	***P*** **-value**	**OR**	**95% CI**
1	556	8.19(7.59,8.96)	8.15(7.49,8.92)	8.29(7.74,9.09)	0.122	1.196	(0.990,1.445)
3	556	9.40(8.48,10.43)	9.09(8.35,9.96)	10.40(9.48,10.46)	<0.001	2.764	(2.137,3.576)
7	556	7.59(6.96,8.54)	7.34(6.84,7.85)	9.08(8.30,9.93)	<0.001	3.753	(2.984,4.720)
14	531	7.13(6.66,7.78)	7.07(6.55,7.52)	7.96(7.08,9.00)	<0.001	2.375	(1.926,2.928)
21	511	6.88(6.42,7.37)	6.83(6.38,7.15)	7.28(6.63,8.32)	<0.001	2.361	(1.860,2.998)
28	476	6.75(6.31,7.16)	6.63(6.27,7.07)	7.08(6.38,7.88)	<0.001	2.330	(1.768,3.069)

*BPD, bronchopulmonary dysplasia; IQR, Interquartile range; OR, Odds ratio; CI, confidence interval. All NT-proBNP values were converted to logarithm and presented as median (IQR)*.

**Figure 3:**
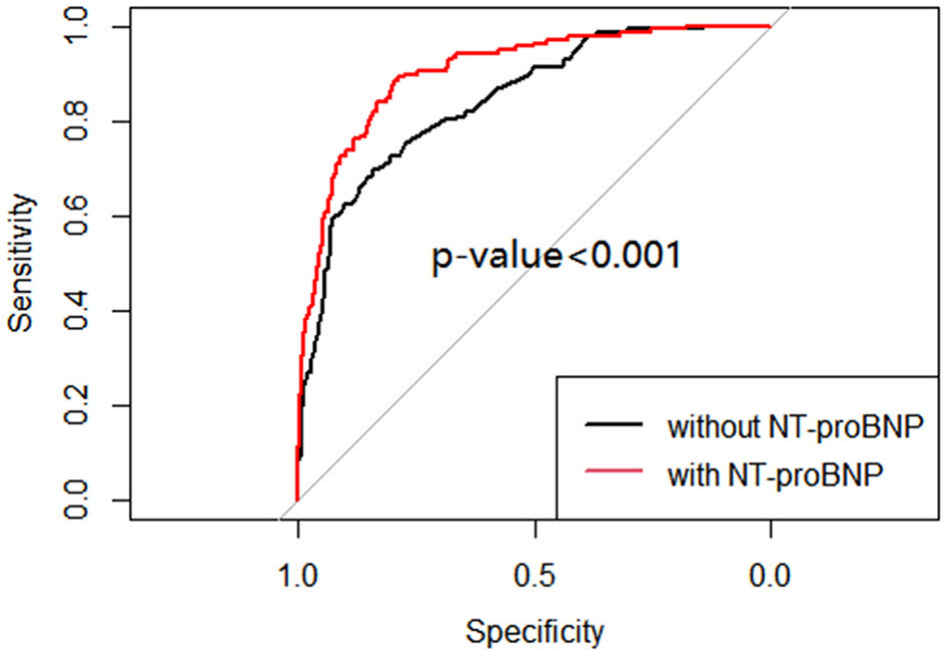
ROC curve of multivariable logistic regression of moderate-to-severe BPD/death with and without NT-proBNP level.

MV and respiratory support patterns are risk factors for BPD (23). Consistent with previous studies, patients in group 2 received a longer MV time in the first week of life and higher respiratory support patterns on the 7th day (±2 days) than those in group 1 (*P* < 0.001). No significant difference was found in the use of caffeine between the groups 1 week after birth (*P* > 0.05; Table 3). After adjusting the risk factors (GA, BW, 1- and 5-min Apgar scores, history of abnormal pregnancy, fetal distress, IUGR, use of surfactants, HsPDA, early PH, MV time, respiratory support pattern) determined by univariate analysis, the NT-proBNP level on the 7th day (±2 days) was still an important predictor of moderate-to-severe BPD/death (OR = 3.152; 95%CI: 2.347~4.234; *P* < 0.001; Table 4). The AUCs of the two ROC curves were 0.846 (0.809, 0.882) and 0.906 (0.877, 0.934) (*P* < 0.001), which showed that the level of NT-proBNP on the 7th day after birth (±2 days) greatly improved the predictive ability of moderate-to-severe BPD/death (Figure 3).

**Table 3:** Comparison of respiratory support and caffeine use in 1 week after birth of both study groups.

	**All patients (*n* = 556)**	**No-to-mild BPD (*n* = 418)**	**Moderate-to-severe BPD/death (*n* = 138)**	***P*** **-value**
Days of MV,1st week [median (IQR)]	0(0,4)	0(0,2)	4.5(0,7)	<0.001
Type of respiratory support of Day7				<0.001
None (%)	82(14.7)	82(19.6)	0(0)	
Warm box/Low-flow nasal cannula (%)	39(7.0)	38(9.1)	1(0.7)	
NIPPV (%)	299(53.8)	247(59.1)	52(37.7)	
Invasive-MV (%)	136(24.5)	51(12.2)	85(61.6)	
Caffeine, 1st week (%)	322(57.9)	241(57.7)	81(58.7)	0.830

*BPD, bronchopulmonary dysplasia; NIPPV, non-invasive positive pressure ventilation; MV, mechanical ventilation*.

**Table 4:** Fully adjusted multivariable estimates of association between NT-proBNP values at day 7 of life and moderate-to-severe BPD/death^a^.

**Predictor**	**B**	**OR**	**95%CI**	***P***
GA	−0.206	0.814	(0.669,0.990)	0.04
Male	0.766	2.172	(1.257,3.753)	0.005
IUGR	1.329	3.776	(1.603,8.899)	0.002
NT-proBNP7	1.148	3.152	(2.347,4.234)	<0.001
Days of MV, 1st week	−0.372	0.690	(0.567,0.839)	<0.001
Type of respiratory support of day 7	2.551	12.817	(4.325,37.989)	<0.001

*BPD, bronchopulmonary dysplasia; NIPPV, non-invasive positive pressure ventilation; MV, mechanical ventilation*.

a *The multivariate model started with all the significant predictors in Table 1 and Table 3. The model went through the stepwise selection by keeping only the statistical significant variables in the final multivariate model*.

**Figure 4:**
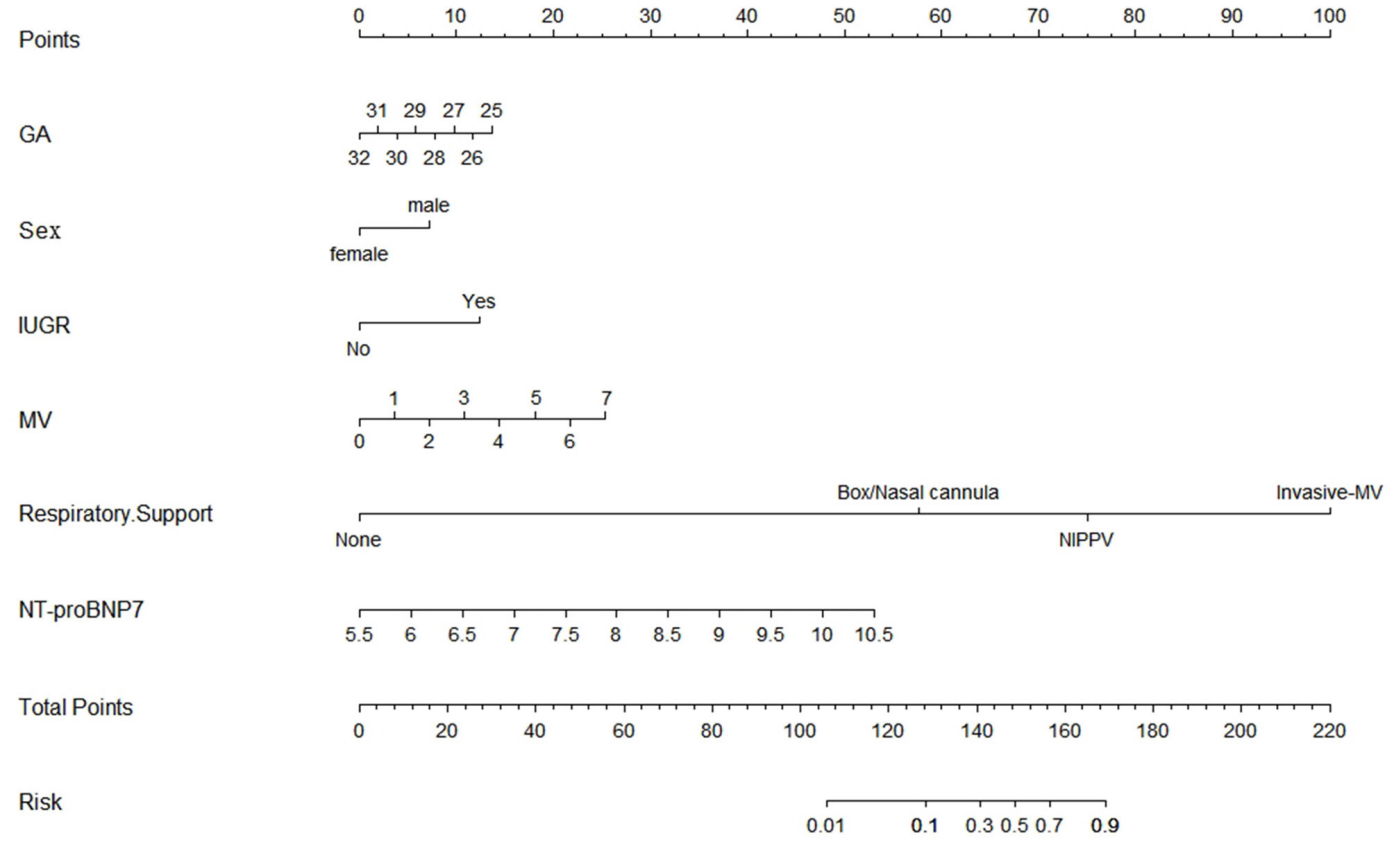
Nomogram for moderate-to-severe BPD or death.

**Figure 5:**
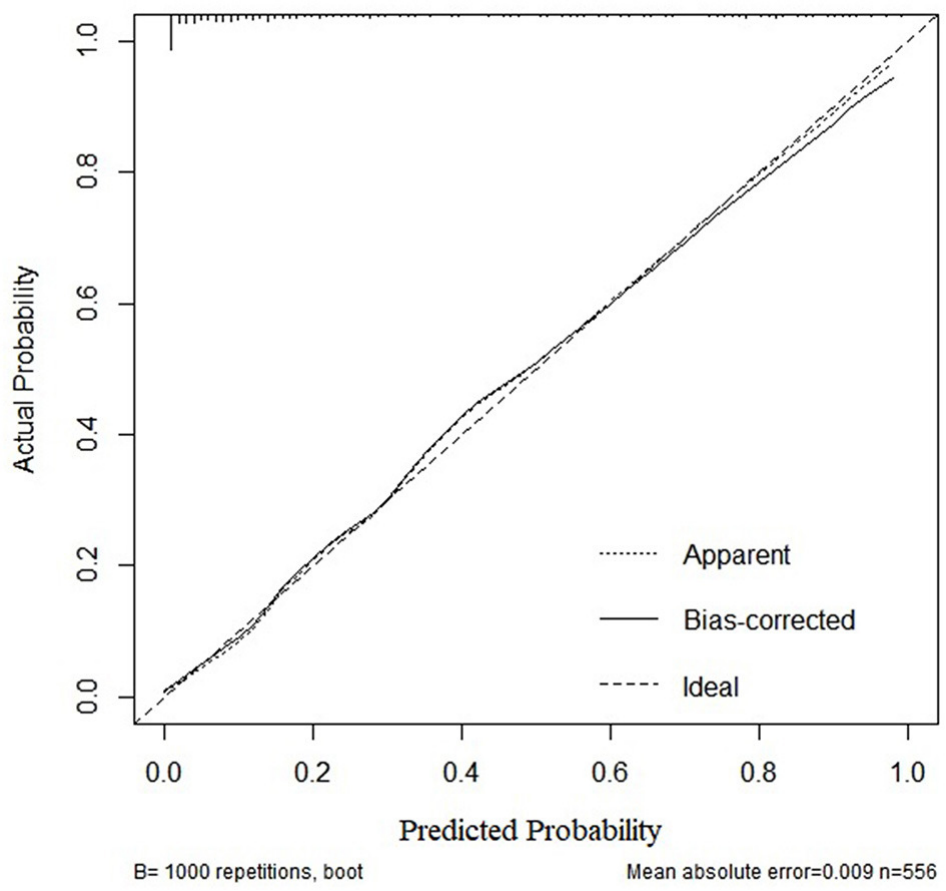
Calibration curves for the nomogram of moderate-to-severe BPD or death.

Based on the multivariate logistic regression and ROC curve results, our study constructed a good model of the early prediction of moderate to severe BPD/death, including the NT-proBNP level. To show the model more easily and intuitively, we used six variables with *P* < 0.05 to construct a risk prediction nomogram (Figure 4). The calibration curve for the nomogram indicated good consistency in this cohort (Figure 5).

## Discussion

To our best knowledge, this study was the first to dynamically evaluate the correlation between the NT-proBNP levels and moderate-to-severe BPD/death in the first 28 days of life, and it had the largest sample size in the study of the correlation between NT-proBNP and BPD. Moderate-to-severe BPD or death was used as the outcome rather than moderate-to-severe BPD because death is a competing outcome with BPD (24). Our results showed that the serum NT-proBNP level of infants with moderate-to-severe BPD/ death was significantly higher than that of infants without/mild BPD from 3 to 28 days after birth, and the predictive value on the 7th day after birth (±2 days) was the highest (the cutoff value was 3360 ng/L, the sensitivity was 80.0%, and the specificity was 86.2%) in VLBWs. Moreover, it remained statistically significant after controlling for confounding variables, including GA and other factors. We also developed a simple and convenient nomogram to predict moderate-to-severe BPD/death that helped clinicians make better treatment decisions and increased the possibility of early intervention for high-risk patients.

In the past few decades, many predictive models for BPD have been developed using clinical variables, such as GA, BW, sex, IUGR, MV time, and HsPDA (25, 26). As expected, smaller GA, male sex, IUGR, MV time in the first week of life and type of respiratory support on the 7th day after birth were important predictors of moderate-to-severe BPD/death, and patients with HsPDA were more likely to develop moderate-to-severe BPD. These known risk factors have increased neonatal pediatricians' awareness of the potential risk of BPD in preterm infants, but the recognition of high-risk patients with moderate-to-severe BPD was limited, making early intervention difficult for specific patients who are most likely to develop the most severe BPD grade and indicating the need for more reliable parameters to improve the accuracy of prediction. Increasing evidence supports the use of the NT-proBNP levels as a biomarker for screening, diagnosis, management and prognosis of heart disease in children (27, 28). Consistent with Kulkarni et al.'s study (29), NT-proBNP levels increased mostly on the 3rd day after birth in our study, because the transition from fetal circulation to neonatal circulation in the first few days of life is accompanied by an increase in pulmonary blood flow and systemic vascular resistance caused by pulmonary dilatation, and a rapid increase in the NT-proBNP levels can reduce the increase in left ventricular load and support myocardial function (30, 31). The serum NT-proBNP concentration of healthy newborns gradually stabilizes in the third month after birth (32). If neonatal diseases such as HsPDA (33), congestive heart defects (34), diaphragmatic hernia (35) and anemia (36) occur, the NT-proBNP level will remain at a high level.

Many studies have used the serum NT-proBNP levels as a potential biomarker of respiratory diseases, including BPD in premature infants (37, 38). However, the mechanism of the increase in NT-proBNP in BPD is not completely clear. In adult studies, plasma NT-proBNP can be used as a biomarker of the exacerbation of chronic obstructive pulmonary disease (39). Our previous studies have shown that NT-proBNP can be used to predict ventilator weaning failure in premature infants with RDS, and a high level of NT-proBNP corresponds to a high rate of extubation failure (40), increasing the risk of BPD (41). The changes in the plasma NT-proBNP levels suggest changes in intrauterine and extrauterine hemodynamics in children. The increase in the plasma NT-proBNP levels in patients with BPD may be related to increased pulmonary vascular pressure, chronic lung disease, diastolic dysfunction or impaired left ventricular function (42). The development of BPD destroys alveolarization and microvascular development, which increases the resistance of pulmonary vessels and may lead to the occurrence of BPD-related PH (43, 44). In premature infants, excessive pulmonary pressure in the immature lungs is accompanied by the continuous maturation of the alveolar and vascular structure, leading to the abnormal development of pulmonary blood vessels (45). At the same time, the response of pulmonary vessels to oxygen may be enhanced, and mild hypoxia can also lead to a significant increase in pulmonary artery pressure (46). Many studies have shown that children with PH have higher levels of NT-proBNP (47–50). In addition to postnatal factors, experimental studies support epidemiological findings that prenatal causes not only short-term abnormalities in respiratory function, but also persistent destruction of the lung and pulmonary vascular structure throughout infancy (such as IUGR). Thus, adverse intrauterine stimulation is sufficient to damage vascular growth and induce long-term and severe PH (51, 52).

A recent prospective study of 229 premature infants suggested that the precursors of atrial natriuretic peptide (ANP) levels measured on the 7th day of life (±2 days) were associated with combined outcomes of BPD or death in univariable models but not after adjusting for cofactors (53). Both ANP and BNP belong to the natriuretic peptide family. ANP is released from atrial myocytes in response to atrial wall stretching and is mostly collinear in children and adults (54). Although ANP is unstable, its secretion can be estimated by measuring the mid-regional pro-atrial natriuretic peptide (MR-proANP). Thus, future studies can add valuable information concerning MR-proANP as a predictor of the occurrence and severity of BPD.

Our study has several limitations. Our data were collected at a single center and may not represent all BPD patients. Additionally, we excluded patients with hereditary metabolic disorders, multiple malformations, severe congenital heart disease and renal insufficiency. Thus this study could not be extended to these specific populations. To measure NT-proBNP, the NT-proBNP value higher than the quantitative upper limit (>35,000 ng/L) was changed to 35,010, but the real value may be much higher than this value, producing a higher average value of NT-proBNP than that calculated in this study. Additionally, this is a relatively mature cohort for BPD, and the results may be different in infants with a smaller GA. In the future, a prospective study involving a larger sample size, multicenter, and internal and external validation is needed to incorporate NT-proBNP measurements into the study of predicting the outcome of preterm infants.

## Conclusion

The level of NT-proBNP on the 7th day after birth (±2 days) can be used as a valuable early biomarker for predicting moderate and severe BPD/death, and the established nomogram can help clinicians predict the risk effectively and easily. Our findings warrant a larger prospective study to include NT-proBNP measurements in predicting the outcomes of VLBWs.

## Data Availability Statement

The raw data supporting the conclusions of this article will be made available by the authors, without undue reservation.

## Ethics Statement

The studies involving human participants were reviewed and approved by the Medical Ethics Committee of the First Affiliated Hospital of Zhengzhou University (Ethical code: 2019-KY-95). Written informed consent to participate in this study was provided by the participants' legal guardian/next of kin.

## Author Contributions

QZ, MS, and ML conceptualized and designed the study, drafted the initial manuscript, reviewed, and revised the manuscript. WD, CL, ZS, and XC designed the data collection instruments, collected data, carried out the initial analyses, and revised the manuscript. LW, FM, PX, and MW coordinated and supervised data collection and critically reviewed the manuscript for important intellectual content. WC, JG, and JZ designed the tables and figures of this article. QZ made a great contribution to the design and implementation of the whole study. All authors approved the final manuscript as submitted and agree to be accountable for all aspects of the work.

## Conflict of Interest

The authors declare that the research was conducted in the absence of any commercial or financial relationships that could be construed as a potential conflict of interest.

## Publisher's Note

All claims expressed in this article are solely those of the authors and do not necessarily represent those of their affiliated organizations, or those of the publisher, the editors and the reviewers. Any product that may be evaluated in this article, or claim that may be made by its manufacturer, is not guaranteed or endorsed by the publisher.
